# Characterization of the Mycovirome of the Phytopathogenic Fungus, *Neofusicoccum parvum*

**DOI:** 10.3390/v13030375

**Published:** 2021-02-27

**Authors:** Armelle Marais, Chantal Faure, Gwenaëlle Comont, Thierry Candresse, Elodie Stempien, Marie-France Corio-Costet

**Affiliations:** 1 Univ. Bordeaux, INRAE, UMR BFP, 33140 Villenave d’Ornon, France; chantal.faure@inrae.fr (C.F.); thierry.candresse@inrae.fr (T.C.); 2 INRAE, UMR SAVE, ISVV, Labex Cote, 33140 Villenave d’Ornon, France; gwenaelle.comont@inrae.fr (G.C.); marie-france.corio-costet@inrae.fr (M.-F.C.-C.); 3 Laboratoire Vigne, Biotechnologies et Environnement (LVBE, EA3991), Université de Haute-Alsace, 68000 Colmar, France; elodie.stempien@gmail.com

**Keywords:** mycovirus, high throughput sequencing, fungi, grapevine trunk disease, *Botryosphaeriaceae*, *Neofusicoccum parvum*

## Abstract

*Neofusicoccum parvum* is a fungal plant-pathogen belonging to the family *Botryosphaeriaceae*, and is considered one of the most aggressive causal agents of the grapevine trunk disease (GTD) Botryosphaeria dieback. In this study, the mycovirome of a single strain of *N. parvum* (COLB) was characterized by high throughput sequencing analysis of total RNA and subsequent bioinformatic analyses. Contig annotations, genome completions, and phylogenetic analyses allowed us to describe six novel mycoviruses belonging to four different viral families. The virome is composed of two victoriviruses in the family *Totiviridae*, one alphaendornavirus in the family *Endornaviridae*, two mitoviruses in the family *Mitoviridae*, and one narnavirus belonging to the family *Narnaviridae.* The presence of the co-infecting viruses was confirmed by sequencing the RT-PCR products generated from total nucleic acids extracted from COLB. This study shows that the mycovirome of a single *N. parvum* strain is highly diverse and distinct from that previously described in *N. parvum* strains isolated from grapevines.

## 1. Introduction

Grapevine trunk diseases (GTDs) are one of the most detrimental diseases of grapevine and a subject of major concern for the viticulture sector worldwide [1]. GTDs consist of three main diseases, Eutypa dieback, Esca, and Botryosphaeria dieback, caused by complexes of fungal pathogens [2,3]. Among them, several species belonging to the family *Botryosphaeriaceae* (Ascomyceta) have been associated with Botryosphaeria dieback, the most common in France being *Diplodia seriata*, *Diplodia mutila*, and *Neofusicoccum parvum* [2,4]. The latter is considered the most aggressive, with *Lasiodiplodia viticola*, forming an internal canker by colonizing the cells and tissues of grapevine wood [5,6,7]. The fungal infection can also lead to foliar necrosis and chlorosis, and finally, causes decline and possibly death of the infected plants [8]. 

Several studies have investigated the interactions between *Botryosphaeriaceae* species and grapevine cultivars and identified some virulence factors involved in the pathogenesis process, pointing out differences between *Botryosphaeriaceae* species [9,10,11,12,13]. Moreover, some studies have highlighted intra-species differences in virulence, showing that there is no clear and strict correlation between the genetic clustering of isolates and their virulence [7,10,14] 

Mycoviruses, or fungal viruses, are widespread in all major fungal taxa, and their presence could be one of the factors involved in the virulence variability observed within some *Botryospheriaceae* species. Indeed, even if many mycoviruses are often considered to have no effect on their hosts’ biology or fitness, some have proved to have a significant impact on the biological properties of the fungal host [15]. In particular, several viruses which confer hypovirulence to the infected strains have been described in *Botryosphaeria dothidea*, an important phytopathogenic fungus [16,17,18]. Moreover, the study of Hu et al. [19] provides insights into the molecular mechanisms underlying the hypovirulence of *B. dothidea* in pear caused by infection by two mycoviruses, botryosphaeria dothidea chrysovirus 1, and botryosphaeria dothidea partitivirus 1 [18,19]. Research on mycoviruses infecting *Botryosphaeriaceae* species that are pathogenic to grapevine is less advanced, but a few viruses have been described, in particular in *Neofusicoccum luteum, N. parvum*, and *D. seriata* [20,21,22]. Two mycoviruses from *N. luteum* have been characterized—neofusicoccum luteum mitovirus 1 and neofusicoccum luteum fusarivirus 1—which belong respectively to the families *Mitoviridae* and *Fusariviridae* [20,21]. The study by Nerva et al. [22] allowed the identification of a novel endornavirus from *D. seriata* (diplodia seriata endornavirus 1) and of six novel viruses infecting *N. parvum* and belonging to the families *Chrysoviridae* (neofusicoccum parvum chrysovirus 1), *Mitoviridae* (neofusicoccum parvum mitovirus 1 (NpMV1)), *Narnaviridae* (neofusicoccum parvum narnavirus 1 (NpNV1), neofusicoccum parvum narnavirus 2 (NpNV2)), and *Botourmiaviridae* (neofusicoccum parvum ourmia-like virus 1). Another identified virus, neofusicoccum parvum RNA virus 1, remains unclassified [22]. However, the impact of these viruses on the biological traits of their hosts has yet to be investigated.

In the present paper, we investigated the virome of an *N. parvum* isolate from Chardonnay grapevine plants collected in Burgundy (France) in 2009, and we report the discovery and the complete or near-complete genome sequences determination of six novel mycoviruses belonging to four different viral families.

## 2. Materials and Methods

### 2.1. Neofusicoccum parvum Isolate COLB Identification and Growth Conditions

*N. parvum* COLB (S-116) was recovered in 2009 from Chardonnay plants showing a decline in nurseries (Burgundy region, France). It was cultivated on malt agar medium (MA, 20 g/L malt, 15 g/ agar) at 22 °C as previously described [9]. After three days at 22 °C on MA medium covered with a film (Hutchinson, Chalette/Loing, France), the mycelium of the isolate was scraped and freeze-dried. DNA was extracted as described previously [23]. Then, after centrifugation, DNA was precipitated in isopropanol at −20 °C, and the pellet washed with ethanol (70%) and then solubilized in ultra-pure water.

Fungal identification was obtained by amplifying and sequencing the Internal transcribed spacer region (ITS) as previously described [23].

### 2.2. Total RNA Extraction, High Throughput Sequencing, and Data Sequence Analysis

Total RNAs were extracted from growing mycelium using the TRI Reagent method (Sigma-Aldrich, Lyon, France). Briefly, fresh mycelium collected from a half plate was ground in the presence of liquid nitrogen and sterile sand in a precooled mortar. The powder was transferred to 2 mL of TRI Reagent, homogenized, and incubated at room temperature for 5 min. Further steps were performed according to the manufacturer protocol. Total RNAs were then submitted to a DNAase treatment using the Turbo DNA-free kit following the manufacturer’s recommendations (Ambion, Thermo Fisher Scientific, Illkirch, France). RNAs were then purified and concentrated (RNA clean and concentrator kit, Zymo Research, Ozyme, Saint-Cyr-l’Ecole, France) before being stored at –80 °C. The sequencing library was prepared from rRNA-depleted RNA (Illumina Ribo-Zero rRNA removal kit) and analyzed on the Illumina HiSeq platform (2*150 nt) by Genewiz (Takeley, United Kingdom). After a quality trimming step, the reads were de novo assembled using CLC Genomics Workbench version 11.0. The resulting contigs were then annotated by BlastN and BlastX comparisons with nucleotide and protein GenBank databases, respectively. Sequences were screened for open reading frames (ORF) using ORF finder (https://www.ncbi.nlm.nih.gov/orffinder/) (Accessed on 26 January 2021) either with the standard genetic code or the yeast mitochondrial one. Conserved protein domains were identified in deduced amino acid sequences using the conserved domain search program on the NCBI website (http://www.ncbi.nlm.nih.gov/structure/cdd.shtml) (Accessed on: 26 January 2021) [24].

### 2.3. Confirmation of the Presence of the Detected Viruses

In order to verify the presence of the identified viruses, specific RT-PCRs were performed using primer pairs designed from the generated viral contigs (Table 1) Total nucleic acids (5 µL) were submitted to two-step RT-PCRs as described by Marais et al. [25]. Briefly, after a reverse transcription step, 5 µl of cDNA were amplified using Dream Taq Polymerase (Thermo Scientific, Illkirch, France) and specific primers for each targeted virus (Table 1). The cycling scheme involved forty cycles of 30 sec at 95 °C, 30 sec at the annealing temperature of the primer pair used (Table 1), and 1 min at 72 °C. A final elongation step of 10 min at 72 °C was added. Amplicons were visualized on 1.5% agarose gel and directly submitted to Sanger sequencing (Eurofins Genomics France SAS, Nantes, France). 

Both 5′ and 3′ extremities of NpVV1, NpVV2, NpMV2, NpNV3, and NpMV3 genomes were completed using a Rapid Amplification on cDNA ends (RACE) strategy and internal primers designed from the contigs (Table 1), following kit provider’s recommendations (Takara Bio Europe/Clontech^©^, Saint-Germain-en-Laye, France). Additionally, the 5′ end of NpVV2 and the 3′ end of NpEV1 were also determined. Amplified fragments were directly submitted to Sanger sequencing (Eurofins Genomics France SAS). 

### 2.4. Phylogenetic Analyses

Nucleotide (nt) sequences and deduced amino acid (aa) sequences of viral contigs were included in phylogenetic analyses, together with the corresponding sequences of related viruses. Multiple alignments were performed using the ClustalW program as implemented in MEGA version 7.0 [26]. Phylogenetic trees were reconstructed using the neighbor-joining technique with strict aa distances. Branch validity was evaluated by a randomized bootstrapping (1000 replicates). Genetic distances (p-distances calculated on nt and aa identity) were calculated using MEGA version 7.0.

## 3. Results and Discussion

### 3.1. Mycoviruses Identification from N. parvum COLB

The ribo-depleted RNAs from a culture of *N. parvum* COLB were analyzed by HTS. After quality control and trimming steps, a total of 115,166,554 cleaned reads were used in the bioinformatic analysis. De novo assembly and BLAST-based annotation allowed us to identify long contigs (between 2064 nt and 13,816 nt), showing sequence similarities with known mycoviruses belonging to the families *Endornaviridae* (one contig), *Totiviridae* (two contigs), *Mitoviridae* (two contigs), and *Narnaviridae* (one contig) (Table 2). The mapping of reads on the assembled contigs showed that 47.4% of the reads were viral reads, unevenly distributed between the six contigs representing putative viruses. Contigs 1 and 2, related to mitoviruses, integrated most of the viral reads (93%), whereas the four remaining contigs represented between 0.5% and 1% of the total reads (1–2% of the viral reads, Table 2). 

**Figure 1:**
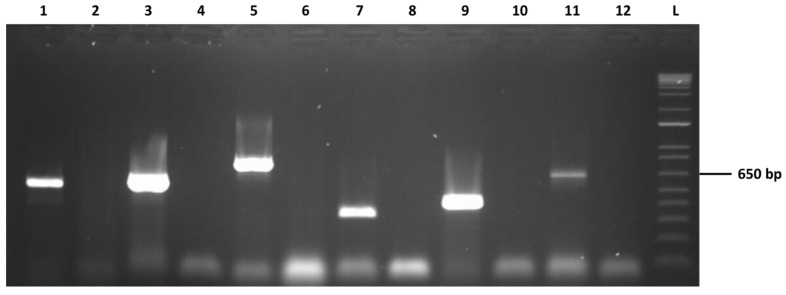
Confirmation of the presence of the six mycoviruses in *Neofusicoccum parvum* COLB by specific RT-PCRs. Primer pairs used are specific for NpMV3 (lanes 1 and 2), NpMV2 (lanes 3 and 4), NpEV1 (lanes 5 and 6), NpVV2 (lanes 7 and 8), NpNV3 (lanes 9 and 10), and NpVV1 (11 and 12). Templates are total nucleic acids from COLB (lanes 1, 3, 5, 7, 9, and 11) and water (lanes 2, 4, 6, 8, 10, and 12). A molecular marker is in lane 13.

The presence of the six mycoviruses was confirmed by RT-PCR experiments using total nucleic acids purified from *N. parvum* COLB mycelium and primer pairs specific for each of the contig (Figure 1). In each case, omitting the reverse transcription step resulted in an absence of amplification (data not shown), demonstrating that the various sequences are only present in unintegrated RNA form in the analyzed total nucleic acid preparations.

### 3.2. Molecular Features and Phylogenetic Relationships of the Identified Mycoviruses

#### 3.2.1. Putative Novel Virus Belonging to the Family *Endornaviridae*

**Figure 2:**
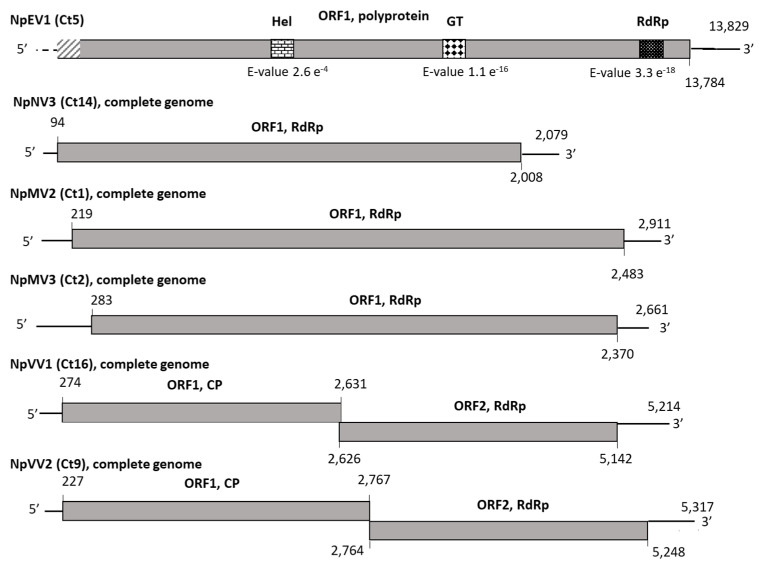
Genomic organization of mycoviruses identified *in Neofusicoccum parvum* isolates COLB. Ct 5, contig 5; NpEV1, neofusicoccum parvum endornavirus 1; Ct 14, contig 14; NpNV3, neofusicoccum parvum narnavirus 3; Ct 1, contig 1; NpMV2, neofusicoccum parvum mitovirus 2; Ct 2, contig 2; NpMV3, neofusicoccum parvum mitovirus 3; Ct 16, contig 16; NpVV1, neofusicoccum parvum victorivirus 1; Ct 9, contig 9; NpVV2, neofusicoccum parvum victorivirus 2. Numbers refer to genomic nucleotide positions. Open reading frames (ORF) are represented by boxes. Conserved RNA-dependent RNA polymerase (RdRp), RNA helicase (Hel), and glycosyl-transferase (GT) domains in the ORF1-encoded polyprotein are represented by striped shading, with the e-values associated with their identification using the conserved domain search program on the NCBI website. Missing sequences are represented by hyphenated lines (in non-coding regions) or dashed boxes (in ORFs).

**Figure 3:**
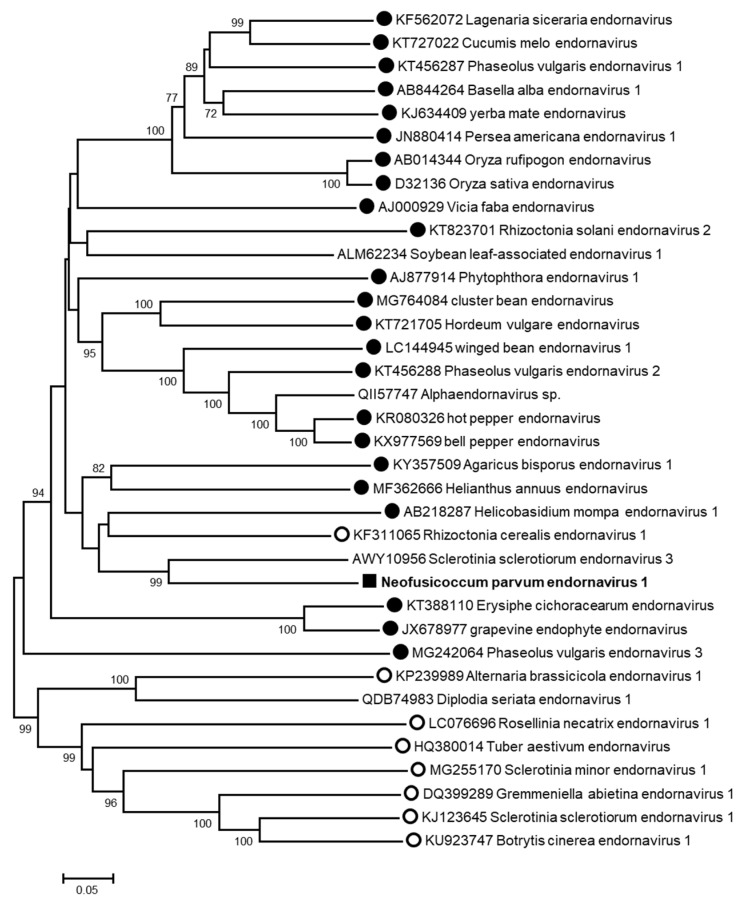
Unrooted phylogenetic tree reconstructed from the alignment of the amino acid sequences of the RdRp domains of *Endornaviridae* representative members. The tree was constructed by the neighbor-joining method and the statistical significance of branches was evaluated by bootstrap analysis (1000 replicates). Only bootstrap values above 70% are indicated. The scale bar represents 5% amino acid divergence. Species belonging to the genera *Alphaendornavirus* and *Betaendornavirus* are identified respectively by a filled or an open circle. Neofusicoccum parvum endornavirus 1 is in bold and indicated by a black square.

Contig 5, showing sequence similarities with *Endornaviridae* members, is 13,816-nt long and comprises a large ORF encoding potentially a polyprotein of 4594 aa (Figure 2) in which several conserved amino acid motifs were identified with high supporting e-values: an RNA-dependent RNA polymerase motif (RdRP_2, cl03049), a glycosyltransferase domain (glycosyltransferase GTB-type, cl10013), and a helicase motif (viral_helicase1, cl26263) (Figure 2). The 3′ end of the genome was determined, whereas the alignment with endornaviral genome sequences suggests that the 5′ end (around 100 nt) is still lacking. To clarify the taxonomic position of this putative endornavirus, a phylogenetic analysis was performed using the RdRp domain of representative *Endornaviridae* members. As shown in Figure 3, this virus, provisionally named neofusicoccum parvum endornavirus 1 (NpEV1), clearly clusters together with members of the genus *Alphaendornavirus*. The amino acid identity between the RdRp domain with that of the most closely related endornavirus, sclerotinia sclerotiorum endornavirus 3, is 63%, whereas the nucleotide sequence identity over the whole contig is 65.9%. The grouping of the RdRp domain sequence in the alphaendornavirus cluster, the presence of a glucosyltransferase domain, and the genome size longer than 11.9 kb strongly indicate that NpEV1 belongs to the genus *Alphaendornavirus* [31]. Moreover, considering the species molecular demarcation criterion accepted in this genus, which is an overall nucleotide identity less than 75% (https://talk.ictvonline.org/ictv-reports/ictv_online_report/positive-sense-rna-viruses/w/endornaviridae/1096/genus-alphaendornavirus) (Accessed on 14 January 2021), we conclude that this virus is a novel species for which the name neofusicoccum parvum endornavirus 1 (NpEV1) is proposed. The near-complete sequence of NpEV1 (13,829 nt) has been deposited in GenBank under the MW175878 accession number.

#### 3.2.2. Putative Novel Viruses Belonging to the Families *Narnaviridae* and *Mitoviridae*

**Figure 4:**
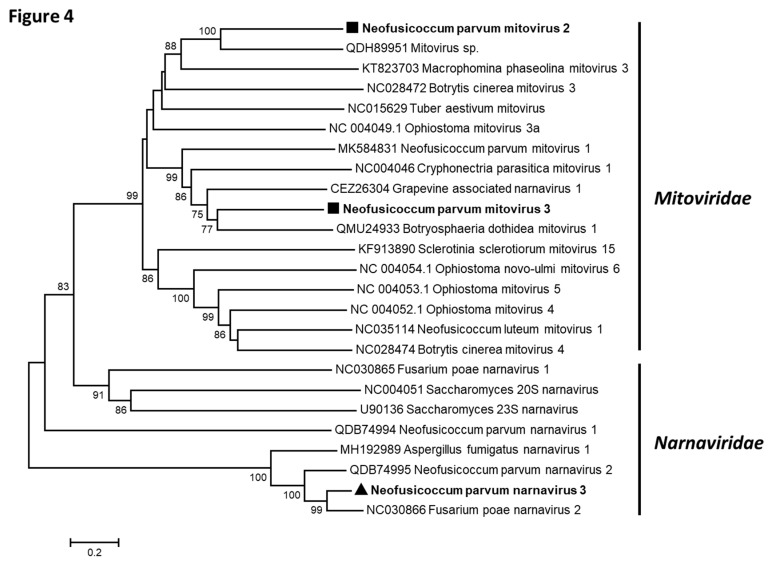
Unrooted phylogenetic tree reconstructed from the alignment of the amino acid sequences of the RdRp of *Narnaviridae and Mitoviridae* representative members. The tree was constructed by the neighbor-joining method and the statistical significance of branches was evaluated by bootstrap analysis (1000 replicates). Only bootstrap values above 70% are indicated. The scale bar represents 20% amino acid divergence. Neofusicoccum parvum mitovirus 2 and 3 are in bold and indicated by black squares, and neofusicoccum parvum narnavirus 3 is in bold and indicated by a black triangle. The families to which belong the species are indicated in the right of the figure.

Three contigs showed similarities with *Narnaviridae* and *Mitoviridae* members (Table 2): Contigs 1 (2885 nt) and 2 (2629 nt) were found to be related to mitoviruses, whereas contig 14 (2064 nt) was more closely related to narnavirus sequences. Both ends of NpMV2 (contig 1), NpMV3 (contig 2), and NpNV3 (contig 14) genomes have been determined by 5′ and 3′ RACE experiments and the complete genomic sequences deposited in GenBank under the accession numbers MW175881-MW175883. As shown in Figure 2, and using the yeast mitochondrial genetic code for contigs 1 and 2, and the standard genetic code for contig 14, each contig comprised unique ORF encoding proteins of respectively 754 aa (NpMV2), 696 aa (NpMV3), and 638 aa (NpNV3) and showing similarities with *Narnaviridae* and *Mitoviridae* RdRp [32]. The taxonomical position of each virus was specified by a phylogenetic analysis based on multiple alignments of amino acid RdRp sequences of representative members of the *Mitoviridae* and *Narnaviridae* families, which comprise only one genus (Mitovirus and Narnavirus, respectively) (Figure 4) [33]. Although belonging to two distinct groups supported by high bootstrap values, NpMV2 and NpMV3 cluster together with members of the genus *Mitovirus*. The putative narnavirus (NpNV3), on the other hand, belongs to the narnavirus cluster. Analysis of the conserved domains of NpMV2 and NpMV3 RdRp sequences revealed the presence of motifs II, III, IV, and VI, in addition to motifs I and V in NpMV3 RdRp, which are typical of mitoviral RdRp [34]. Likewise, the conserved motifs I to V could be found in the NpNV3 RdRp. However, the remaining motifs, especially the GDD motif, are absent, which seems to be a feature of all narnaviruses belonging to this phylogenetic cluster [35]. While a precise molecular species discrimination criterion has yet to be fixed by the International Committee on Taxonomy of Viruses (ICTV), the five distinct *Mitovirus* species so far defined show less than 40% aa identity in their RdRp. NpMV2 and NpMV3 share only 21% aa identity in their RdRp, and at best, only 35.8% (NpMV2 and Mitovirus sp. QDH89952) and 39.6% (NpMV3 and botryosphaeria dothidea mitovirus 1, QMU24933). In particular, they share respectively only 18.8% and 31.4% of aa identity with the mitovirus previously described from *N. parvum* [22]. These elements indicate that NpMV2 and NpMV3 are two novel species in the genus *Mitovirus*, named neofusicoccum parvum mitovirus 2 and neofusicoccum parvum mitovirus 3, respectively.

In the *Narnavirus* genus, only two species have been approved by the ICTV, *Saccharomyces 20S narnavirus* and *Saccharomyces 23S narnavirus*, despite the fact that 71 narnaviruses are reported in the GenBank database, which complicates the determination of the taxonomical position of putative novel species, as it is the case for NpNV3. Nevertheless, sequence criteria (less than 50% aa identity in the RdRp) and biological criteria (stable maintenance of different species in the same host) are proposed for species demarcation [36]. The contrast between the two approved species and the large number of described —but not yet approved—species suggests that a revision of the genus, and possibly of the species demarcation criteria, is overdue. Pairwise comparisons of narnavirus RdRp aa sequences show that NpNV3 RdRp shares more than 50% of aa identity with the corresponding protein of FpNV2 (77.6%), neofusicoccum parvum narnavirus 2 (NpNV2, 67.8%), and aspergillus fumigatus narnavirus 1 (AfNV1, 55.4%) (Table 3). On the other hand, the pairwise aa identity values when comparing FpNV2, NpNV2, and AfNV1 RdRp are comprised between 53.5 % (NpNV2 vs AfNV1) and 67.2% (FpNV2 vs NpNV2). Despite the fact that they have not been officially approved by the ICTV, these three species have been considered as distinct, especially NpNV2 and FpNV2 [22,29]. As a result, it seems that NpNV3 should also be considered, as distinct from FpNV2 and NpNV2 and we propose the name neofusicoccum parvum narnavirus 3 for this novel species.

#### 3.2.3. Putative Novel Viruses Belonging to the Family *Totiviridae*

**Figure 5:**
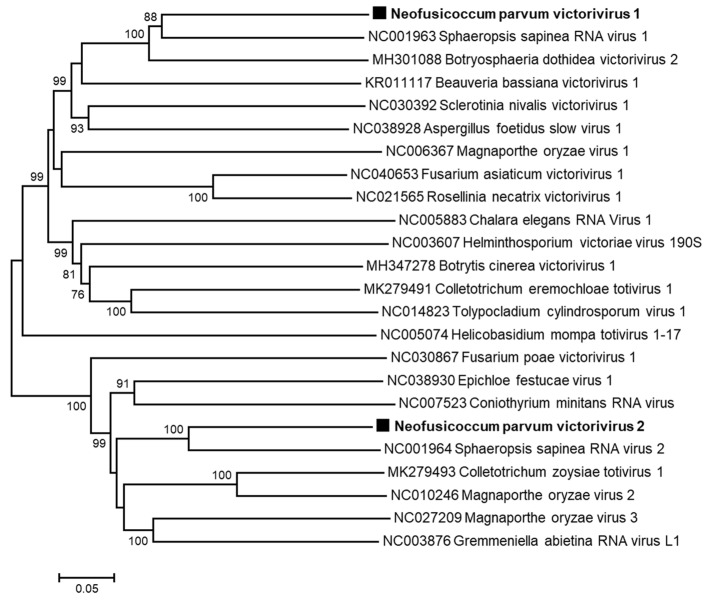
Unrooted phylogenetic tree reconstructed from the alignment of the amino acid sequences of the RdRp of Victorivirus representative members. The tree was constructed by the neighbor-joining method and the statistical significance of branches was evaluated by bootstrap analysis (1000 replicates). Only bootstrap values above 70% are indicated. The scale bar represents 5% amino acid divergence. Neofusicoccum parvum victorivirus 1 and 2 are in bold and indicated by black squares.

The two remaining long contigs that show homologies with viral sequences (Ct9 and Ct16) were found to be more closely related to members of the family *Totiviridae*, and more precisely, to sphaeropsis sapinea RNA virus 2 (SsRV2) and botryosphaeria dothidea victorivirus 2 (BdVV2), respectively, which are a recognized and a tentative species in the genus *Victorivirus*, respectively (Table 2). The 5′ ends of Ct9 and Ct16 were determined, in addition to the 3′ end of Ct16. Both sequences have been deposited on the GenBank database under the accession numbers MW175879 (NpVV1) and MW175880 (NpVV2). Figure 2 shows the genomic organization of the two genomes that comprise two ORFs, with from 5′ to 3′ the ORF1 encoding the putative CP and the ORF2 encoding the putative RdRp [37]. The phylogenetic tree constructed using the alignment of either the RdRp aa sequences (Figure 5) or the CP aa sequences (data not shown) of representative *Victorivirus* members showed that the two putative novel victoriviruses belong to two distinct clusters supported by high bootstrap value. These two viruses share only 35.2% aa identity in the RdRp and 34.6% in the CP (Table 4), which is clearly below the 60% aa identity species demarcation criterion for the genus [38]. As expected for *Victorivirus* members, the CP contains an Ala/Gly/Pro-rich region at the C-termini (data not shown). The relationships of the putative two novel victoriviruses with other genus members were further assessed by RdRp and CP comparisons (Table 4). At the best, NpVV1 shares 62.9% aa identity with the RdRp of sphaeropsis sapinea RNA virus 1 (SsRV1) and 73.9% with the CP of Botryosphaeriaceae dothidea victorivirus 2 (BdVV2). NpVV2 and sphaeropsis sapinea RNA virus 2 (SsRV2) share 66% and 67.2% aa identity in the same gene products, respectively (Table 4). Looking at the same pairwise comparisons performed for various ICTV-approved *Victorivirus* species shows that in several cases, aa identity values higher than 60% are obtained. For example, fusarium asiaticum victorivirus 1 and rosellinia necatrix victorivirus 1, which share 77.6% CP aa identity and 75.3% RdRp identity (Table 4). More examples can be found, with magnaporthe oryzae virus 3 and SsRV1, which share 71.9% CP identity, suggesting that the current 60% CP or RdRp identity species threshold may need to be revised or should not be considered as strict criteria for species demarcation in the genus *Victorivirus*. Based on these observations and on the fact that NpVV1 and NpVV2 and the closest victoriviruses (BdVV2 and SsRV2) were isolated from different hosts, we propose that both victoriviruses characterized from *N. parvum* COLB should be considered novel species and accordingly named neofusicoccum parvum victorivirus 1 and neofusicoccum parvum victorivirus 2, respectively.

## 4. Discussion

In the present study, we report the analysis by HTS of total RNAs from the COLB isolate of *N. parvum*, an important pathogenic fungus involved in the Botryosphaeria dieback of grapevine. Sequences as well as phylogenetic and taxonomical analyses allowed the identification of six putative novel mycoviruses belonging to four different viral families. Two double-stranded RNA viruses, NpVV1 and NpVV2, belong to the genus *Victorivirus* in the family *Totiviridae*. The remaining four viruses are single-stranded positive RNA viruses and belong to the family *Endornaviridae* (NpEV1), the family *Mitoviridae*, with two viruses in the genus *Mitovirus* (NpMV2 and NpMV3) and, the family *Narnaviridae* with one virus in the genus *Narnavirus* (NpNV3). The mycovirome characterized from *N. parvum* COLB is therefore very diverse and different from that recently identified in *N. parvum* isolates from esca asymptomatic and symptomatic grapevines [22]. Indeed, neither endornaviruses, nor victoriviruses were found in those isolates while NpMV2 and NpMV3 are clearly distinct from the mitovirus NpMV1 characterized by Nerva et al. [22]. The taxonomic position of the narnavirus NpNV3 relative to the narnaviruses NpNV2, AfNV1, and FpNV2 [22,29,39] is somewhat less clear. Considering the molecular species demarcation criterion currently accepted for the genus *Narnavirus*, the four tentative species NpNV2, FpNV2, AfNV1, and NpNV3 should be considered as unique species [36]. However, the fact that the two most closely related viruses, NpNV3 and FpNV2, infect different fungal hosts and the fact that a revision of the genus is overdue, both argue in favor of considering NpNV3 as a distinct species. The two victoriviruses characterized here clearly belong to different phylogenetic clusters and are therefore two distinct species. Pairwise comparisons performed with the CP or RdRp sequences from ICTV-approved species suggest that the currently accepted species threshold used (60% [38]) should be re-evaluated in view of the significant number of approved species that do not respect it. As a result, and considering that NpVV1 and NpVV2 and their closest relatives (BdVV2 and SsRV2) were isolated from distinct hosts, we propose to consider NpVV1 and NpVV2 as novel species co-infecting *N. parvum* COLB. 

The characterization of six different viruses from a single isolate indicates that the virome associated with the COLB strain of *N. parvum* is highly diverse, which is a feature shared by other fungal hosts in which rich coinfection patterns have been observed, such as *Fusarium poae* or *Sclerotium rolfsii* [29,40]. Most of the interactions between mycoviruses and their fungal hosts are thought to be neutral for the infected fungi, resulting in symptomless infections [15]; however, in some studies conducted to identify biocontrol agents, it was possible to demonstrate the role of some mycoviruses in modulating the virulence of the infected fungal host, either by increasing or decreasing the aggressiveness, modulating the production of mycotoxins or impacting the growth rate. As an example, a chrysovirus infecting *Alternaria alternata*, a pear pathogen, was shown to have opposite effects on its host, by downregulating its growth rate while enhancing its virulence through stimulation of the production of a fungal effector [41]. The impact of the presence of the six novel mycoviruses on biological traits of *N. parvum* COLB was not investigated here, and it seems to be difficult to predict the outcome of the interactions between the mycovirome and the fungal host, and between the mycoviral species themselves. 

The viral families identified in this study contain viral species that are generally cryptic. Nevertheless, some exceptions exist, with viruses that impact the biological traits of their host. Victoriviruses, members of the family *Totiviridae* are commonly known to be associated with symptomless infections of their hosts [37]. However, the study of Xie et al. [42] reported that infection with the victorivirus helminthosporium victoriae virus 190S induced a hypovirulent phenotype of its natural host, *Helminthosporium victoriae*, but also in a heterologous host. The members of the families *Narnaviridae* and *Mitoviridae* are defined as the simplest of known RNA viruses, encoding no CP and only an RdRp to direct their replication [32]. Members of these two families differ in the site of translation of the RdRp, and probably the site of replication. Members of the family *Narnaviridae* are located in the cytosol, whereas those belonging to the family *Mitoviridae* are confined to the mitochondria of their host cells. Interestingly, the analysis of the virome of culturable fungal endophytes isolated from grapevines reported two novel species of mitovirus and one narnavirus infecting *N. parvum*, without knowing whether these mycoviruses have been characterized from a single isolate [22]. Nevertheless, these results indicate a very high *Narnaviridae* and *Mitoviridae* diversity associated with *N. parvum*. Although most of the mitoviruses and narnaviruses are associated with symptomless infections [32], some exceptions have been described, such as two mitoviruses recently characterized from *Nigrospora oryzae* that can be co-transmitted horizontally and can modulate positively the growth rate of the recipient *N. oryzae* isolate [43]. Hypovirulence related to a mitovirus has also been documented in the phytopathogenic fungus *Sclerotinia sclerotiorum* [44]. Finally, the endornavirus characterized here belongs to the genus *Alphaendornavirus*, which comprises endornaviruses infecting plants, fungi, and oomycetes [31]. They are generally thought to have no obvious effect on their hosts and to be transmitted vertically via host cell division [45]. However, some studies reported the existence of hypovirulence-associated endornaviruses [46,47]. For example, rhizoctonia solani endornavirus 1 (RsEV1) was found to induce metabolic disorders in the infected host, resulting in hypovirulence [46]. Surprisingly, a novel endornavirus from *Sclerotinia minor* (sclerotinia minor endornavirus 1) has been reported to be transmitted horizontally between *S. minor* isolates of different vegetative compatibility [46,47]. 

Co-infections of mycoviruses are common in plant pathogenic fungi, and the determinants of the assembly rules of the mycovirome are not properly understood but are thought to involve multiple factors such as non-self-recognition, RNA silencing, or a role of nutrients or environmental constraints [48,49]. The biology of the co-infections is still very difficult to study and could be very complex, depending on the fungal host, the nature of the mycoviruses, and the interactions between the co-infecting viruses themselves, ranging from synergism to neutrality, antagonism, or mutualism [50]. For example, while both viruses taken individually have no effect on their fungal host *Rosellinia necatrix*, rosellinia necatrix megabirnavirus 2 is able to confer hypovirulence with the help of the co-infecting rosellinia necatrix partitivirus 1, whose accumulation appears to be increased in the co-infected fungal strain [51]. This complexity is also illustrated in *Ceratobasidium sp.* for which the mycelial growth rate is reduced when co-infected with three endornaviruses, while individual endornavirus infections increase the growth rate as compared to the virus-free strains [52]. Further studies are needed to assess the role(s) of each mycovirus identified and of their interactions in the biology, life traits, and fitness of the COLB *N. parvum* strain.

## Figures and Tables

**Table 1:** Primers used in the study.

Primer Name	Targeted Virus (ORF) ^1^	Sequence 5′-3′	Size (nt)	Ta (°C) ^2^	Purpose ^3^
COLB-Endorna-Race3	NpEV1 (3′end)	GGACGGTATGAGACTGACTGGACAGGCG	674	72	RACE
COLB-Alpha-Endorna-FD	NpEV1 (ORF1)	TCCAATGTGGTCCAATGCATC	771	52	Detection
COLB-Alpha-Endorna-RD		GTGTCAACTATTTTTGAAGTG			
COLB-Narna-Race5	NpNV3 (5′end)	TGATTGAAGATGTACAGGGTAAAGA	259	64	RACE
COLB-Narna-Race3	NpNV3 (3′end)	TCATCCTACACATGTGCCATTTAGTGG	168	72	RACE
COLB-Narna-FD	NpNV3 (RdRp)	AAGGGACCTATTGGTTCCGCA	417	58	Detection
COLB-Narna-RD		GGATGTCGATGGACGAAATGT			
COLB-Mito2-Race5	NpMV2 (5′end)	TTCCGTAGAACCGGCATGACACCCACT	141	68	RACE
COLB-Mito2-Race3	NpMV2 (3′end)	TTCGAGCTCTGACTTCGGTCAGTAGGG	361	68	RACE
COLB-Mito2-FD	NpMV2 (RdRp)	AGTCCAACCGTTCCTAAAGAC	568	56	Detection
COLB-Mito2-RD		CGGAATAAGTATAATCAGATCC			
COLB-Mito3-Race5	NpMV3 (5′end)	CCACATCCTGTTGCTCAGACTGCTACAC	444	68	RACE
COLB-Mito3-Race3	NpMV3 (3′end)	CCACAACCTTACTTCCAATCTAACCT	282	58	RACE
COLB-Mito3-FD	NpMV3 (RdRp)	TATTCTTTCACTCCTTGGAGT	538	52	Detection
COLB-Mito3-RD		TCACATAATGAGACCATAACT			
COLB-Victori1-Race5	NpVV1 (5′end)	GCTGGCGGCGGCAAGGGTGGA	128	72	RACE
COLB-Victori1-Race3	NpVV1 (3′end)	GTGACCACGCTACTAATCGAAGCTGGAA	253		RACE
COLB-Victori1-FD	NpVV1 (RdRp)	TCTCCGTCGTTGAGCGGTACCCTG	649	67	Detection
COLB-Victori1-RD		GGTGCTCCGAAGTCATGGGCATT			
COLB-Victori2-Race5	NpVV2 (5′end)	AGGGTAGGCTTGGTGGGAAACGGGAG	326	65	RACE
COLB-Victori2-F5	NpVV2 (RdRp)	AAGCTGAAGCGACATACCGCACG	338	62	Detection
COLB-Victori2-R5		CGAGGCTGAGACGTAAGTGTGTC			

^1^ ORF, open reading frame; RdRp, RNA-dependent RNA polymerase; NpEV1, neofusiccocum parvum endornavirus 1; NpNV3, neofusiccocum parvum narnavirus 3; NpMV2, neofusiccocum parvum mitovirus 2; NpMV3, neofusiccocum parvum mitovirus 3; NpVV1, neofusiccocum parvum victorivirus 1; NpVV2, neofusiccocum parvum victorivirus 2. ^2^ Annealing temperature. ^3^ RACE, Rapid Amplification of cDNA ends.

**Table 2:** Best BLASTX matches of contigs.

Contig Name	Contig Length (nt)	Provisional Virus Name	BlastX Best Hit ^1^ (Accession Number)	Amino Acid Identity	Mapped Reads	% Total Teads	Family	Reference
Ct 1	2885	NpMV2	RdRp Mitovirus sp. (QDH8995)	40.7%	29,121,096	25.3%	*Mitoviridae*	unpublished
Ct 2	2629	NpMV3	RdRp Botryosphaeria dothidea mitovirus 1(QMU24933)	39.8%	22,033,854	19.1%	*Mitoviridae*	unpublished
Ct 5	13,816	NpEV1	Polyprotein Sclerotinia sclerotiorum endornavirus 3 (AWY10956)	55.2%	1,193,076	1.0%	*Endornaviridae*	[27]
Ct 9	5195	NpVV2	CP Sphaeropsis sapinea RNA virus 2 (NP047559)	71.2%	1,143,697	1.0%	*Totiviridae*	[28]
Ct 14	2064	NpNV3	RdRp Fusarium poae narnavirus 2 (YP009272903)	79.4%	598,911	0.5%	*Narnaviridae*	[29]
Ct 16	5194	NpVV1	CP Botryosphaeria dothidea victorivirus 2 (QBA82442)	72.7%	552,571	0.5%	*Totiviridae*	[30]

^1^ RdRp, RNA-dependent RNA polymerase; CP, coat protein.

**Table 3:** Percentage of amino acid identity between representative members of the genus *Narnavirus* in the RdRp protein.

Virus Name ^1^	NpNV3	FpNV2	NpNV2	AfNV1	ScNV-20S	NpNV1	ScNV-23S
**FpNV2**	**77.6 ^2^**						
**NpNV2**	**67.8**	**67.2**					
**AfNV1**	**55.4**	**53.5**	**54.1**				
**ScNV-20S**	7.9	8.2	6.7	8			
**NpNV1**	7.4	7.6	7.7	8.1	8.6		
**ScNV-23S**	7.1	7.4	7.9	6.6	16.8	7.9	
**FpNV1**	5.9	6.1	5.5	6	11.9	9.2	10.7

^1^ NpNV3, neofusicoccum parvum narnavirus 3; FpNV2, fusarium poae narnavirus 2; NpNV2, neofusicoccum parvum narnavirus 2; AfNV1, aspergillus fumigatus narnavirus 1; ScNV-20S, Saccharomyces 20S narnavirus; NpNV1, neofusicoccum parvum narnavirus 1; ScNV-23S, Saccharomyces 23S narnavirus; FpNV1, fusarium poae narnavirus 1. ^2^ Values above 50% are in bold.

**Table 4:** Percentage of amino acid identity between representative members of the genus *Victorivirus* in the RdRp and CP.

Virus Name ^1^	NpVV1	NpVV2	SsRV1 ^2^	BdVV2	SsRV2	FaVV1	RnVV1
**NpVV1**		*34.6%*	***66.3%***	***73.9%***	*34.4%*	*48.8%*	*47.5%*
**NpVV2**	35.2%		*34.6%*	*35.2%*	***67.2%***	*37.9%*	*36.2%*
**SsRV1**	**62.9%**	34.4%		*67.9%*	*36%*	*49.7%*	*49.8%*
**BdVV2**	**60.5%**	34.2%	**60.4%**		*35.8%*	*48.5%*	*48.3%*
**SsRV2**	33.9%	**66%**	35.4%	33.7%		*37.8%*	*37.4%*
**FaVV1**	45%	36%	45.4%	43.5%	35.9%		***77.6%***
**RnVV1**	45%	34.9%	44.5%	43.3%	35.7%	75.3%	

^1^ NpVV1, neofusicoccum parvum victorivirus 1; NpVV2, neofusicoccum parvum victorivirus 2; SsRV1, sphaeropsis sapinea RNA virus 1; BdVV2, botryosphaeria dothidea victorivirus 2; SsRV2, sphaeropsis sapinea RNA virus 2; FaVV1, fusarium asiaticum victorivirus 1; RnVV1, rosellinia necatrix victorivirus 1. ^2^ ICTV approved species are underlined. Amino acid identities in the CP are indicated in italics; values above 60% are in bold

## Data Availability

The sequences reported in the present manuscript have been deposited in the GenBank database under accession numbers MW175878-MW175883.
